# Speaking COVID-19: supporting COVID-19 communication and engagement efforts with people from culturally and linguistically diverse communities

**DOI:** 10.1186/s12889-022-13680-1

**Published:** 2022-06-27

**Authors:** Holly Seale, Ben Harris-Roxas, Anita Heywood, Ikram Abdi, Abela Mahimbo, Ashfaq Chauhan, Lisa Woodland

**Affiliations:** 1 School of Population Health, Faculty of Medicine and Health, University of New South Wales, Sydney, NSW Australia; 2 National Centre for Immunisation Research and Surveillance, Sydney Children’s Hospitals Network, Westmead, NSW Australia; 3 School of Public Health, Faculty of Health, University of Technology Sydney, Ultimo, NSW Australia; 4 Centre for Health Systems and Safety Research, Australian Institute of Health Innovation, Faculty of Medicine, Health and Human Sciences, Macquarie University, Sydney, NSW Australia; 5 NSW Multicultural Health Communication Service, NSW Health and Centre for Primary Health Care and Equity, University of New South Wales, Sydney, NSW Australia

**Keywords:** Pandemic, COVID, Community, Culturally, ethnically, and linguistically diverse, Communication, Engagement

## Abstract

**Background:**

Since the emergence of COVID-19, issues have been raised regarding the approach used to engage with Culturally and Linguistically Diverse (CaLD) communities during this public health crisis. This study aimed to understand the factors impacting communication and engagement efforts during the COVID-19 pandemic from the perspective of crucial CaLD community stakeholders and opinion leaders.

**Methods:**

Forty-six semi-structured telephone interviews were undertaken with key stakeholders who have an active role (established before the pandemic) in delivering services and other social support to CaLD communities in Australia.

**Results:**

Seven key themes emerged: (1) the digital divide and how to connect with people; (2) information voids being filled by international material; (3) Differentiating established with new and emerging communities’ needs; (4) speaking COVID-19; (5) ineffectiveness of direct translations of English language resources; (6) coordination is needed to avoid duplication and address gaps and (7) recognising the improvements in governments’ approach.

**Conclusion:**

Alliances must be set up that can be activated in the future to reduce issues around resource development, translation, and dissemination of messages to minimise gaps in the response. Financial assistance must be provided in a timely way to community organisations to support the development and dissemination of culturally appropriate communication materials.

**Supplementary Information:**

The online version contains supplementary material available at 10.1186/s12889-022-13680-1.

## Background

Populations at risk of COVID-19 infection have been diverse and differ in COVID-19 literacy and social, behavioural, cultural and health practices. In non-pandemic times, people from Culturally and Linguistically Diverse (CaLD) backgrounds may be vulnerable to the factors that contribute to health inequity, including lower health literacy, cultural and language barriers, lower socioeconomic status, lack of provider cultural competence, lack of social support and a sense of disempowerment [1, 2]. During the COVID-19 pandemic, there have been marked racial and ethnic disparities in rates of severe illness and mortality [3, 4]. However, the sources of the COVID-19 differences have been more challenging to analyse compared to establishing the existence of such disparities [5].

Previously it has been suggested that culture can also interplay with virus spread through behavioural and societal variances, including health-seeking behaviour and intergenerational cohabitation [6]. In theory, several factors may heighten the risk of COVID-19 transmission in CaLD communities, including extended family groups living together (large, inter-generational households), higher religiosity, collectivist approaches to childcare and the cultural expectation of family members providing care for each other when sick [7–10]. Several authors have also pointed towards socio-economic factors, including housing arrangements (multi-occupancy living), income, access to insurance and stable housing and occupation [11, 12]. Previous work has also identified that residents located in low-income neighbourhoods are less likely to be able to stay home in response to COVID-19. This can relate to work-related demands [13]. Workers in specific industries, such as meat processing plants, aged care, and hospitality, have been the most impacted by COVID. These jobs tend to be lower-waged and usually comprised of workers from CaLD backgrounds [14]. The Federation of Ethnic Communities’ Councils of Australia (FECCA) reported that ‘40 per cent of skilled migrants still work in lower-skilled jobs’ across Australia [15]. This equates to a higher proportion of people from CaLD backgrounds working in public-facing occupations, including retail, transport or service, occupations where there is little opportunity for physical distancing and higher levels of interaction with people.

In April 2020, letters were sent to federal, state and territory health ministers from 16 critical organisations, including the Ethnic Communities Council of Victoria, Settlement Services International, Migration Institute of Australia, and HOST International, which raised several key concerns about the Australian Governments’ COVID-19 responses. They spoke about a lack of consideration for CaLD communities, including access to appropriate and reliable in-language COVID-19 information and, most specifically, behaviour change advice and directives. Their call to action to the government was to make sure “that CaLD communities are not left behind in their access to and understanding of COVID-19 and are not made more vulnerable than other parts of the Australian community because they have become an afterthought in the rapid response to- curbing infections.” To support enhancements to Australia’s COVID-19 pandemic response, with a particular focus on communication and engagement with CaLD communities, this study aimed to understand the factors impacting pandemic response efforts from the perspective of key CaLD community stakeholders and opinion leaders.

## Methods

Semi-structured in-depth telephone interviews were undertaken with key stakeholders and opinion leaders, of approximately 30–40 min in duration, between January- April 2021. The collective term ‘Culturally and Linguistically Deeeeee (CALD)’ refers to “the non-Indigenous cultural and linguistic groups represented in the Australian population who identify as having cultural or linguistic connections with their place of birth, ancestry or ethnic origin, religion, preferred language or language spoken at home” [16]. The Human Research Ethics Advisory Panel at the University of New South Wales reviewed and approved this study (HC200776). All the methods used in this study were performed per the guidelines outlined by The National Statement on Ethical Conduct in Human Research, published by the Australian Government.

### Sampling

Participants included those who have an active role in delivering services via multicultural health and other support services via migrant resource centres, refugee health services, settlement services, community-based organisations, translation services and primary care settings. In addition, we sought to include people in senior leadership positions in multicultural health and diversity-related activities, whether through advocacy, policy/program development or research. This principally encompassed personnel such as those from government agencies, CaLD community peak bodies/Councils, and CEOs of community organisations.

This study used a range of national-local-personal approaches to recruiting participants. Firstly, an online search of relevant websites was conducted to identify potential participants matching the eligibility criteria. Each potential participant was then contacted via email with an invitation letter. Secondly, interested participants were asked to recommend any colleagues who may be willing to participate directly. Lastly, emails were sent directly to known contacts of the research team working in the relevant sectors. An effort was made to recruit at least one person from Australia’s States and Territories to capture a broad range of views. However, we were unable to recruit any participants from Northern Territory. Participants were included in the study on receipt of informed verbal consent. This study did not collect any identifiable personal information from the participants.

### Data collection and analysis

An interview guide was developed based on a scoping review of the literature and the observations from a research team member working within the multicultural sector. The remaining researchers reviewed the tool (BHR, AH, IA, AM) to ensure that key areas of interest for the study were included. Open-ended questions focused on the following broad topics: perspectives towards the current communication approach being used by the government, factors affecting communication and engagement with CaLD communities, the communication roles and influences of different multicultural services, and suggested options that could be adopted to enhance communication and engagement with CaLD communities around the COVID-19 vaccine program. Additional items and modifications were added iteratively as interviewees raised new relevant issues unforeseen by the study team. All interviews were recorded using a digital recorder and fully transcribed with interviewee consent. Thematic analysis was undertaken using NVIVO 12, informed by a realist approach [17]. The analysis steps included familiarisation (reading the transcripts to understand the meanings conveyed), code generation (identifying significant words, sentences and phrases and organising them into categories), probing for preliminary descriptive themes, and then reviewing and modifying the identified themes. The final stage involved finding associations between themes in order to generate explanations for them. Two authors (HS and AC) read the transcripts, who independently generated initial codes and categories. Any similarities, differences and clustering were noted, and agreement was reached on initial descriptive themes. The descriptive themes were then shared with the remaining research team for further refinement until consensus was achieved.

## Result

Fifty-seven people were contacted to participate, of which 46 interviews were undertaken with key stakeholders and informants across Australia. The characteristics of the interviews are described in Additional file 1 using the CORE-Q reporting format [18].

### The digital divide and how to really connect with people

Before COVID-19, outreach activities with the community were undertaken mainly face to face via caseworkers visiting clients, walk-in appointments/consultations, or group meetings (i.e. with seniors or new mums). Social participation was encouraged: “*language-specific social support groups to citizenship classes, sewing classes, just social classes as well, and lot of information sharing, parenting classes. We had that kiddies’ playgroups and mothers’ groups*” (Interview 1). Following the emergence of localised COVID-19 outbreaks in Australia, participants spoke of the “frantic” need to transfer to virtual or telephone mode to continue the delivery of routine services. This included conducting meetings via teleconferencing platforms to support engagement opportunities and to replace those face-to-face meetings that needed to be suspended. The virtual engagement approaches aimed to ensure that community members received relevant COVID-19 information, including physical distancing measures, lockdown requirements, testing recommendations, and the COVID-19 vaccination program.

Not having access to a computer or the internet, having low digital or English literacy levels, and language barriers were all raised as barriers affecting the community. It was not just the community members who lacked the hardware (computers or modems) or internet access to switch online but also staff members from some community organisations. In these situations, many were heavily reliant on their mobile phones. To support those without the hardware, donated laptops and Wi-Fi dongles were used to ensure case workers and community members could continue to connect. Video conferencing sessions were given to help those in the community who were unsure about how to use Zoom, FaceTime, Viber, WhatsApp or other chat and conference platforms (“*They had taught them how to use some kind of video conferencing so that then they could have their meetings*”). Whereas in other settings, telephone contact continued with clients (i.e., those requiring settlement services) and community members: “*for a lot of the older community, it’s very one-on-one. We implemented the phone checking service where we were identifying the most vulnerable and providing them support and information*” (Interview 12). In one situation, a community organisation started up a Saturday night free call teleconference service to connect women “*who can’t read or write in Somali, but want to know stuff about COVID.”* (Interview 21).

Participants reported a preference for smartphone-based platforms such as Viber, Facebook Messenger, and WhatsApp compared to computer-based platforms such as Zoom. Some organisations started to connect with communities via WhatsApp. However, they reflected that it took time to set up the connections, and the messages were very static.

### Information voids being filled by international material

It was acknowledged that while Australian mainstream media was a good source of disseminating information at large, participants raised concerns that mainstream media may not reach CaLD communities. Participants spoke about “voids or gaps” occurring during the pandemic regarding the availability of COVID-19-related news and government information. These gaps were linked to delays in getting official information translated or materials not being available in all languages. In almost all interviews, participants spoke about the reliance on overseas news programs or the fact that some community members preferred international media that they streamed from their country of origin. Concerns were raised that the information coming from overseas did not reflect the situation in Australia nor the rules/recommendations around COVID-19 pandemic control measures. This issue may have led to some communities misunderstanding their risk of COVID-19. For example, early in the pandemic, there were few COVID-19 cases in Africa, so some community members originating from Africa thought they might be protected from the virus just through their ethnicity. Confusion amongst community members regarding the COVID-19 vaccines, including about which vaccines are recommended or licensed for use, was also linked to CaLD community members watching news channels from abroad.

“I had a conversation with my auntie one day; she watches Greek streaming…, and she was telling me something. I said, “No, that’s not exactly the case because they’re giving us different information here.” She goes, “Yes, but in essence, it’s the same.” She was comfortable receiving the information coming from Greece without having any clue what the Premier was saying, for example, every morning when she was coming out on TV. (Interview 11)

“The other thing was the absolute confusion…It’s complicated to understand full stop, but if you consider that most obviously, everyone in all our communities comes from somewhere else, and most of those places in the world have had a very different experience of COVID. I don’t think that in New South Wales, we were fast enough to provide the messaging to the community, and so we allowed that void to be filled” (Interview 33)

Compounding this issue was some COVID-19 resources, as they included “technical jargon”, which may have been confusing for people with low literacy or health literacy levels, even if English was their first language. Government materials were not available across all languages, nor were they always available in simplified English that was “accessible” to communities.

“*There are some communities that are missing the information. If we take the example of the South Sudanese community…. Dinka is one of the tribes among 64 other tribes. It means you provide information to one tribe; there are 63 missing. That’s what I mean by saying, yes, there’s some information there, but it doesn’t capture everybody. (Interview 1)*.

Participants spoke about interpreting the information to “make it real” in some settings. For some communities, data was unavailable in their local language: “*we can see the Department of Health information flashed in Arabic and Chinese, but no African languages were available”. (Interview 21).* In these situations, it was stressed that community language radio and online information sessions (“Townhalls”) delivered in language were critical. Lastly, there were mixed feelings about Australia’s multicultural and multilingual broadcaster (SBS) role in disseminating information. Some participants described it as brilliant; others were more reserved in their comments and felt it failed to capture what was needed.

“My mother or auntie, there’s no connection to SBS as it used to be years ago. Why would they even go in and watch the Greek news if they can watch it directly from Greece? Why would I wait for the last night’s news if I can watch them live?” (Interview 11)

“Stop wasting money on things like ethnic press and TV. Sure, they do a good job and are important to a degree. Still, theree is always going to be a gap that you’re not going to be able to plug unless the community takes ownership and gets them to do the communication themselves to the people they are close to”. (Interview 40)

One suggestion that was put forward was to have community leaders talk in the language during the news programs on mainstream media channels or for messages to pop up. However, others felt this was still missing the mark.

### Differentiating established with new and emerging communities’ needs

There was not a consistent picture when it came to identifying which community groups should have support/resources directed to. Some participants suggested that newly emerging communities (migrant/refugee) would be most at risk of missing out on messages and support services. One key factor contributing to this was the focus on providing translated COVID-19 information to “high volume” community groups versus “high need” groups. Another issue identified was that established communities have ‘infrastructure’ in the form of community organisations, community leaders, in-language newspapers, radio stations etc. *“I think “it’s the newly arrived communities with minimal infrastructure that are the most vulnerable. Particularly in small communities like the Rohingya, who have only had a written language since the ‘80 s, and hardly anybody can read those”. (Interview 16).* However, not all participants agreed with this sentiment.

Participants also highlighted that Governments did not consider those individuals who come from cultural groups with oral traditions or who are illiterate in their language: “*One of the first outbreaks I think in our region was in the meat works, and most of the employees at the meat works work from Karen and Burmese heritage. Many people in those communities don’t read or write in their own language either, so thanks to the local community leaders, we set up a testing site, and most people heard of it from word of mouth. That’s how, but we do have Karen information written, but we also had some videos made*”. Having verbal messages was identified as being critical. In some suburbs, organisations resorted to door-knocking to encourage people to go and get tested.

The ability to navigate websites was also emphasised as an issue for some community members. Especially at the beginning of the pandemic, it was not easy to locate relevant information on the government’s websites. While participants acknowledged improvements, challenges remained for those community members who did not read in English. Even if online information was translated, there was often the requirement to browse the website in English to find the relevant language: “*The really common accessibility issues were languages were ordered. You had to browse in English alphabetical order. Look, most people know their language’s name in English, but you also need to know the other language”.* Participants noted that not all the COVID-19 resources available online were translated into every language.

### Speaking COVID-19

Issues around low levels of understanding about COVID-19 (about transmission, testing requirements, vaccination program etc.) and increasing amounts of misinformation triggered some organisations to offer online community forums. These forums were provided for interpreters, case workers, and community leaders and often featured bilingual GPs and government officials to offer updated COVID-19 information and answer any questions. In some settings, these sessions were held weekly or fortnightly.

*“We’ve had GPs; we even had an orthopaedic surgeon present weekly COVID updates to audiences, getting the accurate advice, wash your hands, keep your distance, do all that, and talk about the specifics in the community.”* (Interview 18)

Other forums were set up to provide updates to local community leaders so they could pass on relevant information to their communities: *“We established what we called the Greater Western Sydney Community Leaders Forum. What we did was we invited community leaders from across Western Sydney. We had Zoom meetings, initially weekly ones, and we started bringing in the police, for example, to talk about the fines and what was legal or not, and health practitioners to talk about the virus. Then we brought in some tax agents to talk about work, the job seeker and job keeper sort of stuff.” (Interview 11).*

Whilst resource kits were available from the government to support outreach efforts, criticisms were raised that the kits were just information. What was lacking during the early part of the pandemic were training sessions to support the adoption of the resource kit. As one participant indicated, there was a need to provide examples of what other organisations or community groups were doing. Lastly, participants also identified that there was a need to offer training forums to case and settlement workers, as well as translators/interpreters, to support their understanding about COVID-19: *“We ran workshops called Speaking COVID. We’ve focused on engaging with the interpreters in workshops…. We did those bi-language group, and we had some content that talked about what does airborne transmission mean and what’s a droplet, what does isolation mean, and does quarantine mean, and all of the terms that became really common in that period and explained those in ways that the interpreters could understand so that they could, in turn, interpret them appropriately for the clients.” (Interview 16).*

### Ineffectiveness of direct translations of English language resources

Understanding and acknowledging the different cultural beliefs about illness and COVID-19 was critical to developing resources. However, in many settings, participants spoke about the fact that COVID-19 education resources were often created in English and then translated. Potentially as an outcome, participants talked about mistrust and misunderstanding amongst community members, linked to the fact that resources were not tailored to their beliefs or practices: *One story that someone told me is the true story of an older woman in a smaller community who contracted the virus. In that culture, when someone’s sick…you go and visit them*”. (Interview 18). The problem is that the education resources did not account for these practices. Other participants spoke about the words/phrases that were in translated materials that were nonsensical or gave the wrong message: “*when we hear the media or the government saying, ‘We’ve produced the written material, you have to be careful on what you’re saying because no one’s vetted the accuracy of that material. That’s where I’m suggesting that it seems on the surface that the right thing’s been done, but when you look at the content, it’s highly questionable*” (Interview 13). As a solution, it was suggested that education resources be developed from scratch with the targeted communities, which are not only language-specific but also count for the nuances within the community. The videos should include local faces and phrases known in the community”.

“*In other words, you end up with 20 slightly different documents. They’re not all the same, but that’s okay, as long as the basic information is the same, the way it’s presented is slightly different*”. (Interview 17).

Beyond having resources developed in collaboration with the target community, there is still a need to have bicultural workers available to help with supporting community members’ understanding of the information. For example, it was recommended that people avoid ‘share-plates’. However, it is common practice for many Arabic-speaking and African communities to sit and eat communally, with everyone taking food from the main platter. Therefore, bicultural workers were critical in helping break down the messages and reassuring the community that they could still have dinner with their direct family members. These workers do not necessarily have a health background, but they can speak the language and read and write in English and their language. They are seen as having some influence and visibility within their community. In some settings, training was provided to the bilingual workers to become the ‘face’ of the COVID-19 communication strategy, working in partnership with the health officials.

“*We paid our bilingual educators to make calls to people, the networks they had, give them targets, and say, “Try and call at least ten women that you can.” (Interview 43)*

They also enabled two-way feedback and the capacity to hear from the community members regarding any ongoing issues or factors impacting public health strategies such as testing. While participants spoke of the value of these workers, in reality, not all geographic areas had these workers available.

“*We hired eight bilingual workers in various language groups so that they can speak to callers to a particular language-specific phone number to ask for guidance on COVID-related matters. Not to be a substitute for the COVID helpline, but to be able actually to speak to someone in your language,*”. (Interview 13)

### Coordination is needed to avoid duplication and address gaps

The issue of duplication was raised in terms of the development of resources and videos for different community groups: “*What you ended up having was this website with collections of stuff in different languages often saying the same thing, but slightly different. It was a disaster*” (Interview 33). Participants spoke about confusion amongst their community members when there were differing regulations across the different states/territories. They also cited confusion when organisations didn’t “sing from the same songbook” or when messages were not consistent: “*the service providers might send them something, Department of Health sends them something, and then their workplace sends something else, so it creates a lot of misunderstanding, but it creates a lot of confusion for the young people” (Interview 4).*

To reduce these issues, one participant suggested that it would be great to have a “*one-stop-shop where GPs and community members also could access accurate health information according to language that they could easily search up and share with other members of the community*”. In putting for this suggestion, they acknowledge that a considerable amount of work was being done to develop resources but that they were not always stored in an easily accessible format. They were identifying the different needs of communities and the funding available, which needed to be promoted one state to introduce a community connector advisor, who was tasked with getting organisations to talk to each other. Other participants also endorsed the need for coordination at a state level, who felt that coordination of efforts could not be done at the district level: *“Coordination is key… It’s essential to have functioning networks with good information-sharing and healthy relationships to avoid that”. (Interview 14).*

However, duplication was not always framed as having a negative impact on pandemic efforts as in some instances, having multiple videos outlining the public health requirements or promoting the COVID-19 vaccine may be helpful as each community organisation will ‘add their own jargon” and will have a close relationship with the community they serve (especially important in places with solid regionalism). As one participant stated: “*In Queensland, we have quite strong regionalism, and people in the region are sometimes sick of being told what to do by Brisbane all the time. The decisions are from Brisbane in an urban area, and they don’t always understand what’s happening*”. It was acknowledged that if bottom-up approaches like this are going to be done, health experts must also be involved to ensure the messages are accurate.

### Recognising the improvements in governments’ approach

Participants raised concerns that early in the pandemic, the Australian and state governments did not seem to have plans for multicultural communities. Issues were raised with governments, but there wasn’t the sense that organisations were ‘heard’. Participants acknowledged that the government was only “*15 min ahead of us in terms of the decisions and duration of the roll-out, in terms of the policy. A lot was happening, and it was swift. They just didn’t take their time, and maybe they didn’t have that time*. (Interview 12). At a regional level, local government organisations undertook rapid consultations with community members to understand their perceptions of the pandemic and their concerns. Using this information, they could influence the local district response, including the visual and digital resources.

“*The advantage of this was because the community came on board in terms of what should go in, how should it be said, we were able to address the immediate fears and concerns of communities. We were able to have their people as the front face so that we could get the messages across. (Interview 43).*

Two events were signalled as the triggers for the governments revising their approaches. The first event was the hard lockdown in nine public housing towers in inner Melbourne, Victoria. There were significant delays in preparing and distributing materials about the lockdown in community languages and an absence of interpreters. The second event was the issue regarding the translation of COVID-19 materials and the mix-up in languages, including one document with mixed Farsi and Arabic words. As one participant indicated, at that point: “the Department of Health said, “Well, okay, let’s get this done properly and listen.” Following those events, COVID-19 advisory groups focused on multicultural communities, bringing together academics, practitioners, GPs, and community members.

This sense of a shift in the government’s approach from mid-2020 was a consistent message across all the interviews. As one participant suggested, “*they very clearly heard the message from communities, which is stopped doing it to us and start doing it with us*”. Representatives from different community organisations spoke about meeting with federal ministers/policy advisors via Zoom meetings. While there appeared to be improvements, issues were still raised regarding the responsiveness of the different government teams, including the policy versus communication teams.

*My criticism of the Department is that the policy side of the policy team is switched on, and they’re with it; they’re listening to the advice we’re giving them. We’re on the same page. Whereas the communication team, we keep saying the same thing over and over and over again. In the meetings, they say, “Yes, that’s a good idea, and we’ll go away”, and we just keep seeing the same mistakes come up. We see things as fundamental as ensuring that English material is in plain simplified English before it goes to the translators. I think I’ve said that about 12 times. (Interview 40)*

In the initial phase, funding was made available however it was directed towards peak bodies instead of going directly to the community. It was on the peak bodies to organise the distribution of funds via multicultural COVID-19 community grants, which local organisations could apply for to support local activities. For example: “*one community ended up getting some funding,… the women were keen to watch the news and find out what happened, but the news is all in English. They had someone who was taking the announcements… three or four days’ worth and putting it into one chunk of text and having that translated and then shooting that out *via* WhatsApp or WeChat or something, one of those platforms, to all of the members of the community group*. (Interview 16). Lastly, criticisms were made of the funding that was directed towards consultants and creative agencies, as opposed to giving the money to the community to produce locally tailored content: “*just give the money to the communities. They know the communities best, they know what the issues are. It’s targeted, they’re very local.”* (Interview 40).

## Discussion

By undertaking interviews with stakeholders involved in providing support to people from CaLD backgrounds, we were able to gain a rich understanding of the critical challenges encountered around communication and the attempts being used to engage with community members regarding the COVID-19 public health measures and restrictions. Our study results echo the concerns raised by multicultural networks and consumer council reports published since the start of this pandemic [19–21]. The remainder of this paper will focus on consolidating and reflecting on the critical lessons in order to inform ongoing pandemic efforts and revisions to pandemic plans/guidelines. These lessons will centre on three key areas: (1) partnerships and governance processes, (2) supporting community ambassadors, and (3) funding support and mechanisms of distribution.

### Partnerships and governance processes

Throughout this pandemic, the issues of information/resource overload and duplication of efforts have been contrasted with issues regarding community members’ ability to access culturally tailored resources and in relevant modes of delivery. One possible strategy to reduce these issues in future events is to develop a management plan to support the response efforts focused on CaLD communities. A similar document is available to help the emergency response management and operations focused on Aboriginal and Torres Strait Islanders [22]. It outlines the critical partners’ roles and responsibilities, including federal, state and territory governments and sector support organisations. Importantly, it outlines the need for coordination, with emphasis placed on identifying appropriate ways to engage with sector organisations and community stakeholders, establishing meetings for regular updates and sharing of important information, and establishing systems to build trust. Lastly, it also recognised the need to fund dedicated surge capacity to support relevant workers. The need for an advisory group was established early and included Public Health Medical Officers and leaders from the Aboriginal Community Controlled sector; Aboriginal Health Services; state and territory government public health and medical officials; Aboriginal communicable disease experts; the Australian Indigenous Doctors’ Association; and the National Indigenous Australians Agency.

In comparison, the need for a dedicated and tailored management plan for CaLD communities has not been historically outlined. Coming into this COVID-19 pandemic, Australia’s response was traditionally guided by the Australian Health Management Plan for Pandemic Influenza (AHMPPI) [23], which was last updated in August 2019. This document acknowledged the need for Australia’s public health response to be guided by the need to ensure equity in providing care and recognise the cultural values and religious beliefs of different community members. However, beyond that reference, the only other acknowledgement of the need to bring in other sector parties was linked to the delivery of the pandemic-specific immunisation program, with the recognised need to have education sessions delivered by CaLD community groups. With the emergence of COVID-19 came the release of an updated response plan, the Australian Health Sector Emergency Response Plan for Novel Coronavirus (COVID-19 Plan) [24]. The document was published early in the pandemic and only referenced the need for potential support to be provided to remote and rural communities (including remote and rural Aboriginal and Torres Strait Islander communities) if needed. However, it took until December 2020 until an advisory committee was constituted focused on CaLD communities [25]. The advisory committee was constituted for the duration of need (with an extension currently given until the end of 2021). It had three separate working groups focused on communication, vaccination, and data. The purpose of the advisory group was to advise the Department of Health on the ‘experience of culturally, ethnically and linguistically diverse people and communities about the COVID-19 pandemic’. Looking beyond the COVID-19 pandemic, there is a need for a national advisory board to be created earlier in pandemics and large-scale emergencies to support meaningful partnerships and streamline the dissemination of communication and resources in a timely way. It could also have greater oversight into how funding is used to support communication efforts and signal to governments where barriers remain. The need for a national advisory group has been recently echoed by others [26].

At a state or district level, the value of having an interagency collective approach was outlined in a report focused on the network response that occurred in Queensland [27]. The initial core group was based on pre-existing collaboration between hospitals, health services, primary health networks and refugee health networks, with additional partners representing key multicultural agencies, settlement services, community councils, and government. This informal interagency network aimed to engage with all stakeholders and work with Queensland Health to facilitate COVID-19 public health messaging. An evaluation of the interagency collective approach highlighted that a strong partnership between the agencies has been formed and that there was a high level of satisfaction, despite the time requirements [27]. Regarding deliverables, the interagency collective demonstrated significant inputs and outputs as part of the collective response, including regular meetings, community and leader information/training sessions, newsletters and translated resources. One of the partners involved with the collective response stated, “*We have achieved a great amount collectively in an ever-changing environment with multiple players and complexities. This could not have been achieved without the partnership approach*.” [27]. However, it was acknowledged that more work was needed to clarify and formalise partnership structures and processes. While these partnerships may have also been in place in other settings around Australia, to our knowledge, this was the largest, representing a broad scope of partners. Understanding the enablers and the structures and strategies that could strengthen the partnership would assist other States in introducing these collectives for both pandemic and non-pandemic emergencies.

### Supporting community-based ambassadors

The need to support CaLD community leaders, faith leaders, and multilingual and settlement sector workers to be able to communicate about COVID-19 was a key message that came through all the interviews. These practitioners have been described as the “conduit between the Government and communities” based on their role in ensuring that messages are accessible, meaningful, and effective for communities [19]. The need to provide opportunities for education and training was identified early by some of the stakeholders we interviewed and acted on, with virtual meetings organised by local health districts and state health departments. But there was certainly no real sense whether these information/training sessions were available across all States/Territories or consistently delivered to all sectors.

The need to provide the latest information on COVID-19 and medical and community resources to community leaders was highlighted in an article by Panagis Galiatsatos and colleagues [28]. The article outlined a program that commenced in early March 2020 for faith community leaders, which involved twice-weekly 60-min conference calls [28]. Beyond providing information to participants, the sessions also allowed the leaders to voice concerns and ask questions. As the pandemic continued, the spectrum of participants extended to representatives from religious communities, senior centres, hospitals and other health care centres, community service organisations, and the local government. Beyond supporting the understanding around COVID-19 of those on the calls, the authors also identified that the information from the community calls was being “*shared by phone calls, texts, and e-mails. Other participants have shared the information with caregiver support groups, book clubs, community associations, Sunday school classes, and colleagues*” [28].

Beyond ensuring community leaders and other stakeholders can receive the latest guidance about the situation and ask questions, there is also the need to consider broader training opportunities during pandemics or other emergencies, as well as ways to expand the potential pool of practitioners rapidly in response to pandemics and health emergencies [29, 30]. Potential focus areas for training could be around strategies that support communication, discussing vaccines and effectively addressing misinformation. Other possible locations could be around developing resources for the community which account for health literacy needs.

‘Community ownership’ was a phrase that was repeatedly used by participants during the interviews, with emphasis placed on engaging communities in the development and testing of messages (and images) and audio/visual materials. The need for bottom-up communication approaches involving stakeholders and tailored materials has been repeatedly echoed in the published literature (and by our participants), as it enhances accessibility, usability, and inclusiveness [26, 31]. A bottom-up approach starts with understanding the targeted community, their information needs and what information/resources will satisfy these needs. This also means engaging different community actors, including the community, faith leaders, and those seen as trustworthy and relevant [32]. However, these processes require funding to support the development and time of the people involved. It is also critical that local public health units work closely with community actors to ensure that the health messages are accurate and reflect recommendations. Issues have been reported during the COVID pandemic regarding the accuracy of statements, the variability in health messages translated by community leaders, as well as situations where the values/beliefs of community leaders have not aligned with the health experts and government policy, and so conflicting messages, for example around the COVID-19 vaccine have been promoted. In some situations, anti-vaccination messages have been sent out by religious leaders [33–35]. To reduce this, it is suggested that health units and governments work closely with the community leaders, whereby community leaders and representatives write the materials for their community. Then the senior medical advisor fact-checks the resource.

Lastly, while participants spoke about the close networks with community leaders and ambassadors, concerns were still raised about how actively governments (federal and state) engage with these actors. Efforts must be taken to explore the feedback mechanisms being used and to ensure that feedback is being collected (and integrated into policy/revisions to strategies) from all of the different CaLD communities, as well as across urban, regional and remote areas. One suggestion is to establish WhatsApp groups with community ambassadors or community volunteers to collect questions, tips, and concerns. However, collecting this information is not enough; it must be discussed and shared across the response sectors.

### Funding support and mechanisms of dissemination

The lack of resources and funding to support local initiatives was raised as an issue by the participants. It is important to note that challenges around funding for the community sector were evident before the COVID-19 pandemic [36]; however, these issues were heightened with some in the industry speaking about the challenges of the ‘inflexible funding’ that was made available and their inabilities to provide appropriate levels of support to community members or to pay staff appropriately. A report focused on the impact of COVID-19 on the community sector identified issues, including the ongoing struggles of organisations to meet the workloads (“working beyond capacity”) and the reliance on volunteers [36]. Without the efforts of community leaders and volunteers, the response would have been grossly inadequate. Based on feedback from community sector workers and organisational leaders, it was suggested that funding needed to be increased from government sources and businesses and philanthropic funders are required to offer more significant financial support to community organisations. Importantly, given the level of uncertainty and change, funding must be flexible.

In planning for future events, there is a need to ensure that consideration of CaLD communities must be incorporated into Commonwealth and state and territory planning. In addressing the specific needs of CaLD communities, recognition must be given to the diversity within and between communities. A one size fits all approach is not likely to be effective, acknowledging that some communities can mobilise their resources and staffing [37]. Our participants spoke about the need to support those communities that were recently established in Australia, whereas others have suggested that CaLD communities living in regional and remote areas need particular attention. Another suggestion is to ensure funding is available to CaLD media outlets.

### Limitations

The study team acknowledges that CaLD communities are distinct yet heterogeneous groups with unique health delivery needs [38]. Efforts were therefore made to ensure stakeholders were recruited across a range of different CaLD communities. However, it should be acknowledged that we could not include participants from all the other CaLD communities in Australia. The following are noted as limitations for this work: (1) interviews were only undertaken with a select group of participants, so the possibility of other important themes emerging cannot be ruled out; (2) the use of snowball recruitment may have also reduced the range of opinions amassed from participants; and (3) specific details regarding the participants’ role was also not collected. However, the concordance across all states and territories and different CALD groups/roles of participants gives credibility to our study results.

## Conclusion

During the COVID-19 pandemic, there have been issues around the poor quality, delays in the materials available to communities, and conflicting messages. There is an urgent need to capture these key lessons and use them to strengthen not only Australia’s pandemic response but that of other countries with diverse communities, to ensure that future responses are equitable, represented and appropriately resourced. Alliances must be set up that can be activated in the future to reduce issues around resource development, translation, and dissemination of messages to minimise gaps in the response. Financial assistance must be provided in a timely way to local organisations to support the development of culturally appropriate communication materials. Not all communities require translated materials, so it is essential that tailoring and targeted approaches are used to ensure those community members who may be more vulnerable in public health events are not left behind.

## Supplementary Information

**Additional file 1.** CORE-Q Consolidated Criteria for Reporting Qualitative Research.

## Data Availability

Available upon request from the primary author.
